# The Role of eHealth Literacy and Patient Adherence in Mediating Health Consciousness and Perceived Severity in Quality of Life Among Young Patients With Ischemic Heart Disease: Cross-Sectional Study

**DOI:** 10.2196/71647

**Published:** 2026-05-26

**Authors:** Puteri Sofia Nadira Megat Kamaruddin, Azmawati Mohammed Nawi, Mohamad Nurman Yaman, Rajiv Chowdhury, Mohd Shawal Faizal Mohamad, Abdul Muizz Abdul Malek, Ezarul Faradianna Lokman, Mohd Fairulnizal Md Noh

**Affiliations:** 1 Nutrition, Metabolism and Cardiovascular Research Centre, Institute for Medical Research, National Institutes of Health, Ministry of Health Malaysia, Shah Alam, Malaysia; 2 Department of Public Health Medicine, Faculty of Medicine, University Kebangsaan Malaysia Medical Centre, Ministry of Higher Education Malaysia, Jalan Yaacob Latif, Kuala Lumpur, 56000, Malaysia, 60 389215555; 3 Department of Medical Education, Faculty of Medicine, Universiti Kebangsaan Malaysia, Ministry of Higher Education Malaysia, Kuala Lumpur, Malaysia; 4 Robert Stempel College of Public Health and Social Work, Academic Health Center 5, Florida International University, Miami, United States; 5 Cardiology Medical Department, University Kebangsaan Malaysia Medical Centre, Ministry of Higher Education Malaysia, Kuala Lumpur, Malaysia; 6 Cardiology Department, Medical, Serdang Hospital, Ministry of Health Malaysia, Kuala Lumpur, Malaysia

**Keywords:** eHealth literacy, patient adherence, quality of life, young ischemic heart disease, chronic disease

## Abstract

**Background:**

Ischemic heart disease (IHD) is becoming increasingly prevalent, with a rising trend significantly impacting the quality of life (QoL) of young Malaysians.

**Objective:**

This study aimed to investigate the direct and indirect relationships between eHealth literacy (eHealth) and patient adherence (PA), as well as their mediating effects on the associations of health consciousness (HC) and perceived severity to chronic disease (PS) with QoL among young patients with IHD.

**Methods:**

A cross-sectional study was conducted at 2 hospitals, recruiting eligible patients through consecutive sampling at outpatient cardiology clinics. Data were collected via a validated self-administered questionnaire encompassing sociodemographic and socioeconomic status, medical history, and PS, HC, eHealth, PA, and QoL data. Structural equation modeling analysis was used to evaluate the relationships. The ethics approval was obtained from the Ethical Committee of Universiti Kebangsaan Malaysia Medical Centre (FF-2021-117).

**Results:**

A total of 136 young patients with IHD participated in the study. Structural equation modeling analysis revealed that eHealth had the strongest positive effect on QoL (β=0.287, *P*=.002), followed by PA (β=0.245, *P*=.02), and HC (β=0.218, *P*=.02). Two significant mediation models were identified, aligning with the Transactional Model of eHealth Literacy theory. The first model demonstrated parallel mediation, where eHealth (β=0.125, *P*=.008) and PA (β=0.083, *P*=.046) significantly mediated the relationship between HC and QoL. The second model indicated serial mediation through eHealth and PA between HC and QoL (β=0.042, *P*=.049). The parallel mediation model exhibited medium predictive power and was deemed the best-fit model.

**Conclusions:**

The parallel model pathway showed significant direct and indirect associations between eHealth, PA, HC, and QoL, with eHealth demonstrating the strongest association. Higher eHealth and PA were associated with QoL among young patients with IHD; interventions to support eHealth warrant further investigation in longitudinal or interventional studies.

## Introduction

### Overview

Cardiovascular diseases (CVDs) are the primary cause of mortality globally, with ischemic heart disease (IHD) serving as the largest contributor to this health burden. According to the World Health Organization (WHO), an estimated 19.8 million people died from CVDs in 2022, representing approximately 32% of all global deaths. Of these, about 85% were caused by heart attacks and strokes, and over three-quarters of CVD-related deaths occurred in low- and middle-income countries [1]. The Sustainable Development Goals established by the United Nations in 2015 comprise 17 interconnected objectives designed to tackle a broad range of social, economic, and environmental issues, with a target for achievement by 2030 [2]. A primary objective of Sustainable Development Goals 3 (good health and well-being) is to achieve a one-third reduction in premature deaths caused by noncommunicable diseases through effective prevention and treatment strategies [3].

Although IHD predominantly affects older adults, its prevalence among younger populations is increasing, particularly in South Asia [4,5]. In Malaysia, research indicates a rising prevalence of IHD among individuals less than 45 years of age, a pattern similarly observed in other Southeast Asian nations. In a study, 6.1% of Malaysian patients with IHD were below 45 years old, with a mean age of 39 (SD 6) years [6]. Another study found that 16% of patients undergoing percutaneous coronary intervention for IHD were considered “young,” defined as men below 45 years of age and women below 55 years of age [6,7]. While IHD is less common in younger individuals, it can result in significant morbidity, emotional distress, and financial burdens, particularly because these individuals are the most productive.

For young patients with IHD, managing the disease is particularly challenging, as they must balance health concerns with work, family, and social responsibilities [6,7]. The recurrence of IHD can drastically reduce quality of life (QoL), leading to physical, emotional, and social difficulties [8]. Understanding and improving patient adherence (PA) to medical recommendations is crucial for managing IHD and enhancing QoL within this demographic [9,10]. Research has shown that good adherence can improve outcomes, whereas nonadherence can lead to disease progression, reduced functionality, and diminished QoL [10,11].

In the modern digital era, young people are increasingly dependent on technology for accessing health information. eHealth, as defined by the WHO, refers to the cost-effective and secure use of information and communication technologies in support of health and health-related fields, including health services, surveillance, and education. Correspondingly, eHealth literacy is the ability to seek, find, understand, and apply health information from electronic sources to address or solve a health problem, which has become vital for effectively managing chronic conditions such as IHD [12,13]. The effect of eHealth literacy on PA and overall QoL in young individuals with IHD remains unclear [14]. While some studies suggest that higher eHealth literacy can improve disease management and adherence, the literature does not provide a clear consensus on this relationship [15,16].

### Theoretical Framework

Despite increasing global interest in eHealth literacy, there remains a theoretical gap in understanding how it translates into behavioral outcomes, such as PA, particularly among younger populations with chronic conditions such as IHD. Existing models often focus on general populations or older adults, leaving the mechanisms linking eHealth literacy to PA and QoL in young patients with IHD insufficiently explored. Moreover, the dynamic and context-sensitive interactions proposed by the Transactional Model of eHealth Literacy (TMeHL) have yet to be empirically tested among younger cohorts with IHD, underscoring the need to bridge this theoretical void.

The TMeHL, proposed by Paige et al [17], conceptualizes eHealth literacy as a dynamic and reciprocal process influenced by personal, social, and technological contexts. It posits that individuals continuously interact with digital health information, shaping their knowledge, attitudes, and health behaviors through iterative exchanges. The model integrates individual determinants, such as health consciousness (HC) and perceived severity to chronic disease (PS), with behavioral outcomes, such as PA, ultimately influencing health and QoL. Guided by this framework, this study applies the TMeHL to examine both direct and indirect (mediated) pathways linking these constructs among young patients with IHD.

Guided by TMeHL, this study sought to examine the connections between eHealth literacy, PA, and QoL in young individuals with IHD in the Klang Valley, an urban agglomeration in Malaysia. From a practical standpoint, there is a regional knowledge gap concerning how eHealth literacy and adherence interact within the sociocultural context of Malaysia and the broader Southeast Asian region. While global literature on digital health and adherence is expanding, few studies have investigated how regional variations, such as differences in health care accessibility, digital infrastructure, and cultural perceptions of health, affect these relationships. Understanding these contextual nuances is vital for developing tailored eHealth interventions and PA strategies relevant to young patients with IHD in Malaysia and neighboring countries. This study investigates how eHealth literacy and PA mediate the relationships between HC, PS, and QoL among young patients with IHD, thereby advancing the understanding of how digital health competencies translate into improved management and QoL. To achieve these objectives, a cross-sectional study design was adopted, using validated self-administered questionnaires to capture multidimensional constructs related to eHealth literacy, PA, and QoL among young patients with IHD.

## Methods

### Study Design, Setting, and Reporting Standards

This cross-sectional study was conducted between November 2021 and June 2022 at 2 tertiary cardiac referral centers in the Klang Valley, Malaysia: Hospital Serdang, Selangor, and Hospital Canselor Tuanku Muhriz, Kuala Lumpur. Both centers provide specialist cardiology services, including cardiac rehabilitation and structured outpatient follow-up, for patients diagnosed with IHD. The inclusion of a public specialist hospital and a university hospital was intended to ensure diversity while maintaining comparable clinical management across sites.

The reporting of this study adhered to established international reporting guidelines to enhance transparency and reproducibility. The manuscript was prepared in accordance with the STROBE (Strengthening the Reporting of Observational Studies in Epidemiology) statement for cross-sectional research (Checklist 1) [18].

### Participants and Eligibility Criteria

The study population comprised young adults aged 18 to 45 years diagnosed with IHD (unstable angina, ST-elevation myocardial infarction [STEMI], non-STEMI, or stable angina) who attended cardiology outpatient follow-up appointments during the study period. Eligible participants were Malaysian patients who were able to understand either English or Malay and had provided written informed consent.

Patients were excluded if they had documented dementia or psychiatric illness, evidence of alcohol intoxication, or altered mental status (eg, lethargy, confusion, restlessness, or delirium) that could impair their ability to participate in study procedures. Eligibility was verified through documented case notes and clinical assessment prior to enrollment.

### Recruitment Procedures and Sampling

Participants were recruited from cardiology outpatient clinics and follow-up services during routine clinical visits. Consecutive sampling was employed, whereby all eligible patients attending the clinics during the recruitment period were approached sequentially until the required sample size was achieved. This approach was selected due to the specialized nature of the young population with IHD and logistical constraints during the COVID-19 pandemic. Participants primarily consisted of patients previously discharged from cardiology wards who were attending scheduled outpatient follow-up or cardiac rehabilitation appointments.

Clinic appointment lists and medical records were screened by the researchers to identify potentially eligible and clinically stable patients. Eligible individuals were approached during their outpatient visits and provided with a written participant information sheet outlining the study objectives and the voluntary nature of participation, and written informed consent was obtained prior to enrollment.

### Data Collection Procedures

Following consent, participants completed a validated self-administered questionnaire that included sections on sociodemographic characteristics, medical history, PS, HC, eHealth literacy, PA, and QoL. Depending on participant preference and prevailing public health restrictions during the COVID-19 pandemic, questionnaires were administered either in printed format during clinic visits or electronically via an online platform. Participants were allowed to seek clarification from the researcher at any time during completion.

### Ethical Considerations

#### Ethics Approval

The ethical approval for this study was obtained prior to data collection from the Ethical Committee of Universiti Kebangsaan Malaysia Medical Centre (FF-2021‐117) and the National Medical Research Register, Ministry of Health Malaysia (NMRR-21-178-57942). Institutional permissions were also secured from the participating hospitals and cardiology departments before recruitment commenced.

#### Informed Consent

Written informed consent was obtained from all participants prior to enrollment. Participants were provided with a participation information sheet outlining the purpose of the study, procedures involved, potential risks and benefits, and their rights as research participants. Individuals were informed that participation was voluntary and that they could decline participation or withdraw from the study at any time without consequences to their clinical care or services received. Contact information for the researcher was provided should participants have any questions or require clarification before consenting.

#### Privacy and Confidentiality

All collected data were treated as confidential. Participants’ identities were protected through the use of unique identification codes, and identifiable information was accessible only to the research team for study purposes. Questionnaires were administered in either printed or secure online formats, and data were stored in password-protected systems accessible only to authorized researchers. Personal health information was handled in accordance with institutional [19] and national ethical guidelines [20] to ensure participant privacy and data security.

#### Compensation

No financial incentives or material compensation were provided to participants for their involvement in this study.

### Hypotheses Development

Guided by the TMeHL and empirical evidence from prior research [17], this study proposed a series of hypotheses to examine both the direct and indirect relationships among HC, PS, eHealth literacy, PA, and QoL among young patients with IHD (MultimediaAppendices 1  2). Specifically, it was hypothesized that both HC and PS would have significant positive effects on QoL (H1-H2). Furthermore, higher levels of PA and eHealth literacy were expected to be directly associated with improved QoL (H3–H4).

In addition to these direct associations, the study also tested the mediating roles of eHealth literacy and PA. It was posited that PA and eHealth literacy would each independently mediate the relationships between HC and QoL (H5-H6), as well as between PS and QoL (H7-H8). Beyond these parallel pathways, the study further hypothesized that eHealth literacy and PA would operate in a serial sequence, such that eHealth literacy enhances PA, which in turn improves QoL, when initiated by HC (H9) and by PS (H10).

Finally, the study postulated that the overall structural model would demonstrate strong predictive capability in explaining the variance in QoL among young patients with IHD (H11).

### Study Tools

Translation and cross-cultural adaptation were conducted to ensure the questionnaire’s content validity and to preserve its psychometric properties [21-25]. A validated and reliable self-administered questionnaire was utilized, as described in a previous study [26].

Part A explored participants’ sociodemographic and socioeconomic characteristics, including age, sex, race, gross monthly income, occupation, and education level. These variables were categorized following the National Health and Morbidity Survey guidelines [27]. Part B collected information about participants’ medical history, such as comorbidities, coexisting medical conditions, and their diagnosis of IHD (unstable angina, non-STEMI, STEMI, or stable angina). The duration since the initial IHD diagnosis was also recorded.

Part C focused on PS via a validated 4-item scale (Cronbach α=0.907) originally developed for South Koreans [28]. PS refers to how an individual personally evaluates the seriousness of a chronic health condition. Part D assessed HC through a 5-item scale addressing various dimensions of health awareness and concern (Cronbach α=0.720‐0.835) [29-31].

Part E assessed eHealth literacy, which is the capacity to find, assess, and utilize eHealth information for managing health [32]. The original eHEALS (eHealth Literacy Scale) in English, which had a Cronbach α of 0.90, was modified for use in this study [33]. Part F examined PA to recommendations and behaviors via a modified English version of the Medical Outcomes Study Specific Adherence Scale. This validated scale (Cronbach α=0.58‐0.77) measures PA to medication regimens and healthy lifestyle practices [34-38].

Part G employed the World Health Organization Quality of Life: Brief Version (WHOQOL-BREF) questionnaire, a widely used tool for evaluating QoL in patients with cardiovascular disease. This self-administered questionnaire consists of 26 questions that evaluate 4 areas: physical health, mental well-being, social connections, and the environment. The WHOQOL-BREF questionnaire (Malay) demonstrates high internal consistency, with a Cronbach α of 0.89 for 24 items [39].

### Sample Size

Sample size determination was conducted using the *F* test of regression via G*Power software (Erdfelder, Faul, and Buchner) [40]. A power analysis for multiple regression with 9 predictors was performed to determine the appropriate sample size. The sample size was determined using a medium effect size, consistent with Cohen convention, to ensure adequate statistical power while maintaining a feasible and methodologically sound sample size in the absence of prior effect estimates [41]. The measure (*f^2^*=0.15) [42,43] was set with an α level of 0.05 and a statistical power of 80%. The minimum required sample size for this study was 114 participants.

### Data Analysis

The analysis was performed using the SPSS software (IBM Corporation, 2019) and SmartPLS version 4.0.9.2 (SmartPLS GmbH) [44], a widely used tool for statistical analysis. Patient characteristics were summarized through the descriptive analysis. Continuous variables were reported as means and SDs, whereas categorical data were presented as frequencies and percentages.

The study used partial least squares (PLS) analysis via SmartPLS version 4.0.9.2 [44] to explore the TMeHL theory. A 2-stage approach was used to examine direct and indirect effects (parallel and serial mediation) and to assess the model’s predictive power in relation to QoL among young patients with IHD [45]. This approach enabled the evaluation of the exploratory nature and applicability of the TMeHL theory within this context.

## Results

### Participant Characteristics

A total of 136 patients with IHD, with a mean age of 39.2 (SD 5.22) years, took part in the study. The majority were of Malay ethnicity (n=98, 72.1%) and male (n=112, 82.4%). Socioeconomically, the participants reported an average monthly income of RM 2447.88 (SD RM 2186.11; approximately US $544.18 [SD US $485.99]), with most being private employees (n=62, 45.6%) and having attained secondary education (n=71, 52.2%). With respect to their medical background, most patients had multiple comorbid conditions (n=95, 69.9%) and were diagnosed with STEMI (n=59, 43.4%) within the past 1 to 5 years (n=79, 58.1%; Table 1).

**Table 1.** Sociodemographic characteristics of young patients with ischemic heart disease (IHD) who participated in a cross-sectional observational study conducted at 2 tertiary cardiac referral centers in the Klang Valley, Malaysia, between November 2021 and June 2022 (N=136).

Participant’s characteristics	Values
Sociodemography	
Age (y), mean (SD)	39.2 (5.22)
Gender, n (%)	
Male	112 (82.4)
Female	24 (17.6)
Race, n (%)	
Malay	98 (721)
Chinese	17 (12.5)
Indian	17 (12.5)
Others^a^	4 (3)
Socioeconomy	
Monthly income (RM), mean (SD)	2447.88 (2186.11)^b^
Occupation, n (%)	
Government employee	16 (11.8)
Private employee	62 (45.6)
Self-employed	32 (23.5)
Unpaid worker/housewife	8 (5.9)
Not working	18 (13.2)
Education level, n (%)	
Nil	9 (6.6)
Primary	6 (4.4)
Secondary	71 (52.2)
Tertiary	50 (36.8)
Medical history	
Comorbid, n (%)	
Nil	41 (30.1)
Yes	95 (69.9)
Diagnosis, n (%)	
STEMI^c^	59 (43.4)
NSTEMI^d^	41 (30.1)
Unstable angina	24 (17.6)
Stable angina	12 (8.8)
Years diagnosed with ischemic heart disease, n (%)	
Less than 1 year	50 (36.8)
1‐5 years	79 (58.1)
6‐10 years	7 (5.1)

a Bumiputera Sabah and Sarawak.

b Approximately US $544.18 (SD US $485.99).

c STEMI: ST-elevation myocardial infarction.

d NSTEMI: non–ST-elevation myocardial infarction.

### Measurement Model

The multivariate normality assumption was tested and found to be violated, as reflected by Mardia multivariate skewness (β=11.567, *P*<.001) and kurtosis (β=73.321, *P*<.001) [46]. Consequently, nonparametric bootstrapping with 5000 samples was performed during the structural model assessment [47]. A full collinearity assessment confirmed the absence of bias from single-source data, with variance inflation factor values ranging from 1.032 to 1.636 (see Multimedia Appendix 3).

The measurement model was evaluated for outer loadings (indicator reliability), convergent validity, and internal consistency reliability (see Multimedia Appendix 4). The outer loadings ranged from 0.668 to 0.937 for all the constructs, exceeding the acceptable threshold of 0.5, and no items were removed. Convergent validity was determined by examining the average variance extracted values, ranging from 0.632 to 0.823, and factor loadings, all above the recommended 0.50 threshold [48]. The values for composite reliability were between 0.792 and 0.965, reflecting strong internal consistency.

The Heterotrait-Monotrait ratio of correlation was subsequently used to evaluate discriminant validity. Table 2 shows the Heterotrait-Monotrait ratio of correlation values ranging from 0.122 to 0.590, meeting the condition of ≤0.85 [47,49], confirming that all the constructs are distinct from each other.

**Table 2.** Discriminant validity using the Heterotrait-Monotrait ratio of correlations among constructs derived from a cross-sectional study examining the relationship between eHealth literacy, patient adherence, health consciousness, perceived severity to chronic disease, and quality of life among young patients with ischemic heart disease attending tertiary cardiac referral centers in the Klang Valley, Malaysia (November 2021-June 2022; N=136), analyzed using partial least squares-structural equation modeling (PLS-SEM).

Variable	Health consciousness	Patient adherence	Perceived severity to chronic disease	Quality of life	eHealth literacy
Health consciousness	—^a^	0.370	0.590	0.376	0.540
Patient adherence	0.370	—	0.228	0.454	0.450
Perceived severity to chronic disease	0.590	0.228	—	0.122	0.425
Quality of life	0.376	0.454	0.122	—	0.410
eHealth literacy	0.540	0.450	0.425	0.410	—

a Not applicable.

### Structural Model

#### Direct Effects

Table S3 in Multimedia Appendix 1 presents the hypothesis testing of direct effects, controlling for variables, such as age, comorbidities, education, sex, IHD diagnosis, years diagnosed with IHD, monthly income, occupation, and ethnicity. These control variables were identified due to their well-established influence on patients with IHD, as reported in previous studies [50,51].

The analysis revealed that HC (β=0.218, *t*=2.114, *P*=.02), PA (β=0.245, *t*=2.123, *P*=.02), and eHealth literacy (β=0.287, *t*=2.887, *P*=.002) were positively associated with QoL, as confirmed by bias-corrected CIs that did not straddle 0. Conversely, PS (β=−.157, *t*=1.543, *P*=.06) was not significantly associated with QoL.

Among the direct relationships with QoL, eHealth literacy exhibited the strongest association (β=0.287), followed by PA (β=0.245) and HC (β=0.218). Together, these variables explained 30.8% of the variance in QoL (*R*²=0.308), indicating that the model accounted for a substantial proportion of the factors influencing QoL in young patients with IHD.

In terms of effect size, eHealth literacy (*f*²=0.075), PA (*f*²=0.066), and HC (*f*²=0.038) had weak but substantive effects on QoL, with eHealth literacy showing the largest effect size. In contrast, PS had the smallest effect size (*f*²=0.025) and was not significantly related to QoL.

#### Parallel Mediation Model

**Figure 1.**
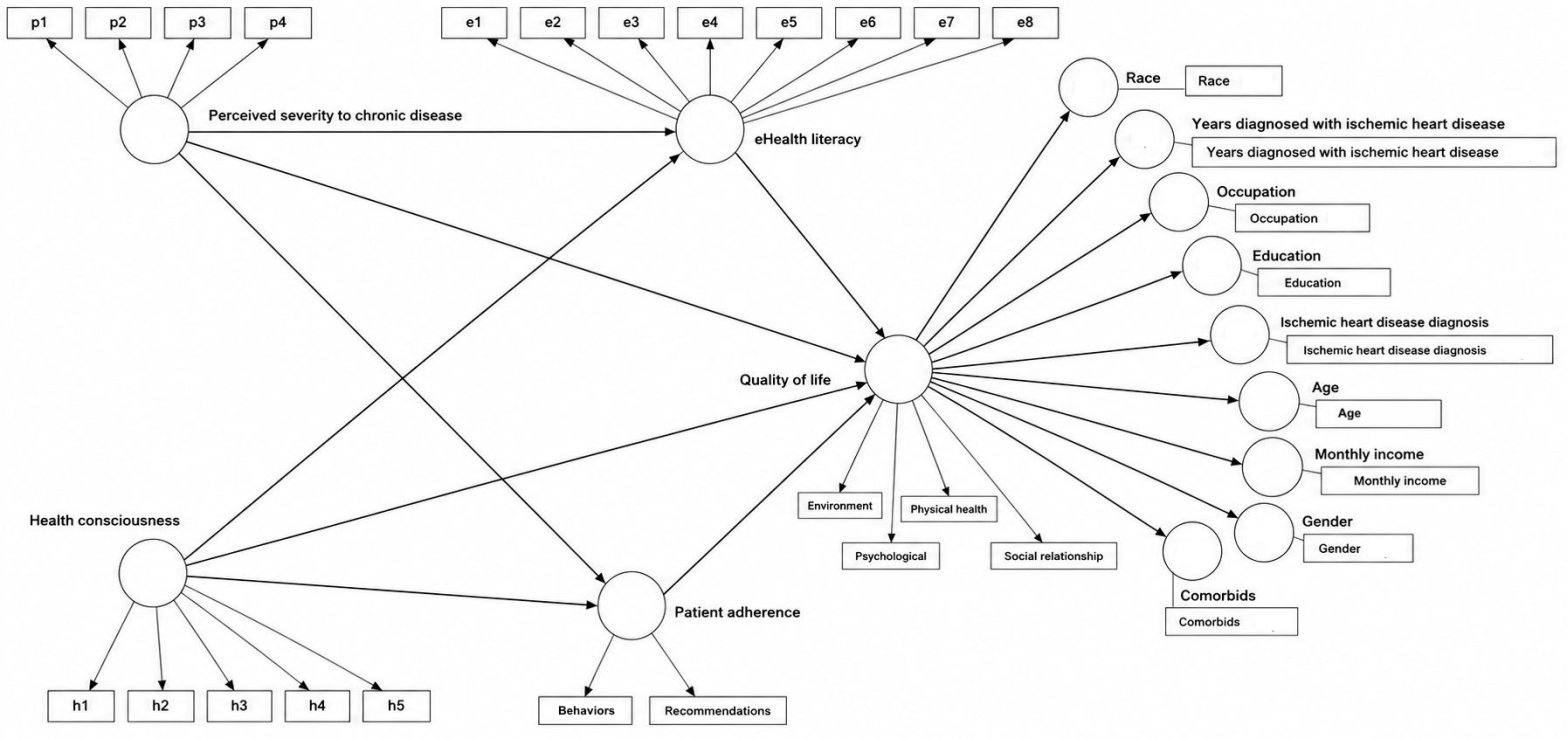
Parallel mediation model evaluated using partial least squares-structural equation modeling (PLS-SEM) in a cross-sectional study among young patients with ischemic heart disease at tertiary cardiac referral centers in Klang Valley, Malaysia (November 2021-June 2022), illustrating hypothesized pathways linking health consciousness and perceived severity to chronic disease with quality of life through eHealth literacy and patient adherence.

Table S4 in Multimedia Appendix 2 and Figure 1 depict that bootstrapping of the indirect effect was significant for both parallel pathways initiated by the construct of HC: (HC → PA → QoL [β=0.083, *t*=1.687, *P*=.046]) and (HC → eHealth literacy → QoL [β=0.125, *t*=2.403, *P*=.008]). The bias-corrected CIs for both the lower limits (BCI LLs) and upper limits (BCI ULs) did not straddle 0, confirming that mediation occurred. Overall, both hypotheses were supported, whereby H5 stated that PA positively mediated the relationship between HC and QoL, with almost no effect size (*f^2^*=0.0069), whereas H6, with a small effect size (*f^2^*=0.0156) of eHealth literacy, positively mediated the relationship between HC and QoL.

Moreover, bootstrapping of the indirect effect for both parallel pathways initiated by PS was not significant, and the hypotheses were not supported. Both H7 (PS → PA → QoL [β=−0.014, *t*=0.461, *P*=.32]) and H8 (PS → eHealth literacy → QoL [β=0.046, *t*=1.219, *P*=.11]) were not positively related to QoL. Both the BCI LLs and BCI ULs straddled 0, confirming that mediation was absent. H7 and H8 both had no effect sizes.

#### Serial Mediation Model

**Figure 2.**
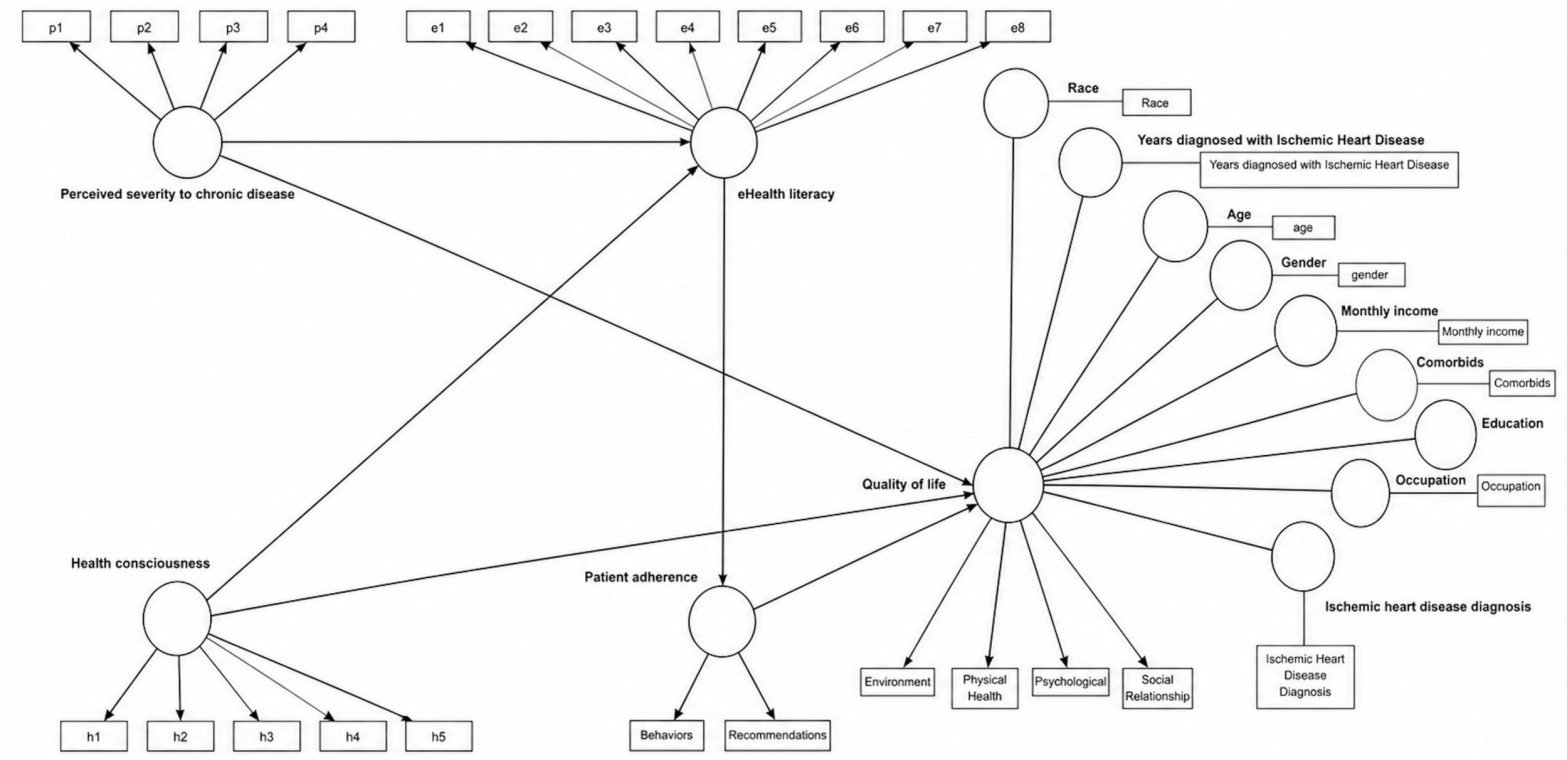
Serial mediation model tested using partial least squares-structural equation modeling (PLS-SEM) in a cross-sectional study of young patients with ischemic heart disease in Klang Valley, Malaysia (November 2021-June 2022), demonstrating sequential mediation pathways involving eHealth literacy and patient adherence influencing quality-of-life outcomes.

Table S4 in Multimedia Appendix 2 and Figure 2 show that the bootstrapping of the indirect effect was significant for the pathway (HC → eHealth literacy → PA → QoL [β=0.042, *t*=1.653, *P*=.049]), but it was not significant for the pathway (PS → eHealth literacy → PA → QoL [β=0.016, *t*=1.037, *P*=.15]). Both the BCI LLs and BCI ULs did not straddle 0, confirming that mediation occurred for H9 but not H10. Overall, H9 supported the hypothesis that eHealth literacy and PA positively and serially mediated the relationship between HC and QoL but with almost no effect size (*f^2^*=0.0018). On the other hand, H10, with no effect size (*f^2^*=0.0003) of eHealth literacy and PA, did not positively mediate the serial relationship between HC and QoL.

#### The Predictive Power of the Model (Out-of-Sample Prediction)

Table 3 illustrates the predictive power of the model via PLS-predict. In this study, PLS-predict was applied exclusively to the construct of QoL, using a 5-fold procedure with 5 repetitions. The Q² values for QoL were 0.078 and 0.010 for the parallel and serial mediation models, respectively. The root mean square error or mean absolute error (MAE) was then compared with the values from a linear model (LM) for each construct item, according to the guidelines of Shmueli et al [52]. While the root mean square error is the default metric [53], the MAE was used in this analysis because the histogram indicated a nonnormal distribution.

**Table 3.** PLS-predict model assessment demonstrating the predictive performance of parallel and serial mediation models derived from a cross-sectional study among young patients with ischemic heart disease attending tertiary cardiac referral centers in Klang Valley, Malaysia (November 2021-June 2022; N=136), using PLS-SEM^a^^b^.

Quality-of-life items	Q² predict	PLS-SEM_MAE^c^	LM^d^_MAE	PLS-LM MAE
Parallel mediation model^e^				
Environment	0.077	11.895	12.004	−0.109
Physical health	0.035	11.277	11.078	0.199
Psychological	0.037	11.984	12.413	−0.429
Social relationships	0.072	16.015	16.042	−0.027
Serial mediation model^f^				
Environment	0.020	12.379	12.004	0.375
Physical health	−0.012	11.621	11.078	0.543
Psychological	0.005	12.293	12.413	−0.120
Social relationships	0.000	16.914	16.042	0.872

a 95% CI was used with a bootstrapping of 5000.

b PLS-SEM: partial least squares-structural equation modeling.

c MAE: mean absolute error.

d LM: linear model.

e This model demonstrated medium predictive power.

f This model demonstrated low predictive power.

**Figure 3.**
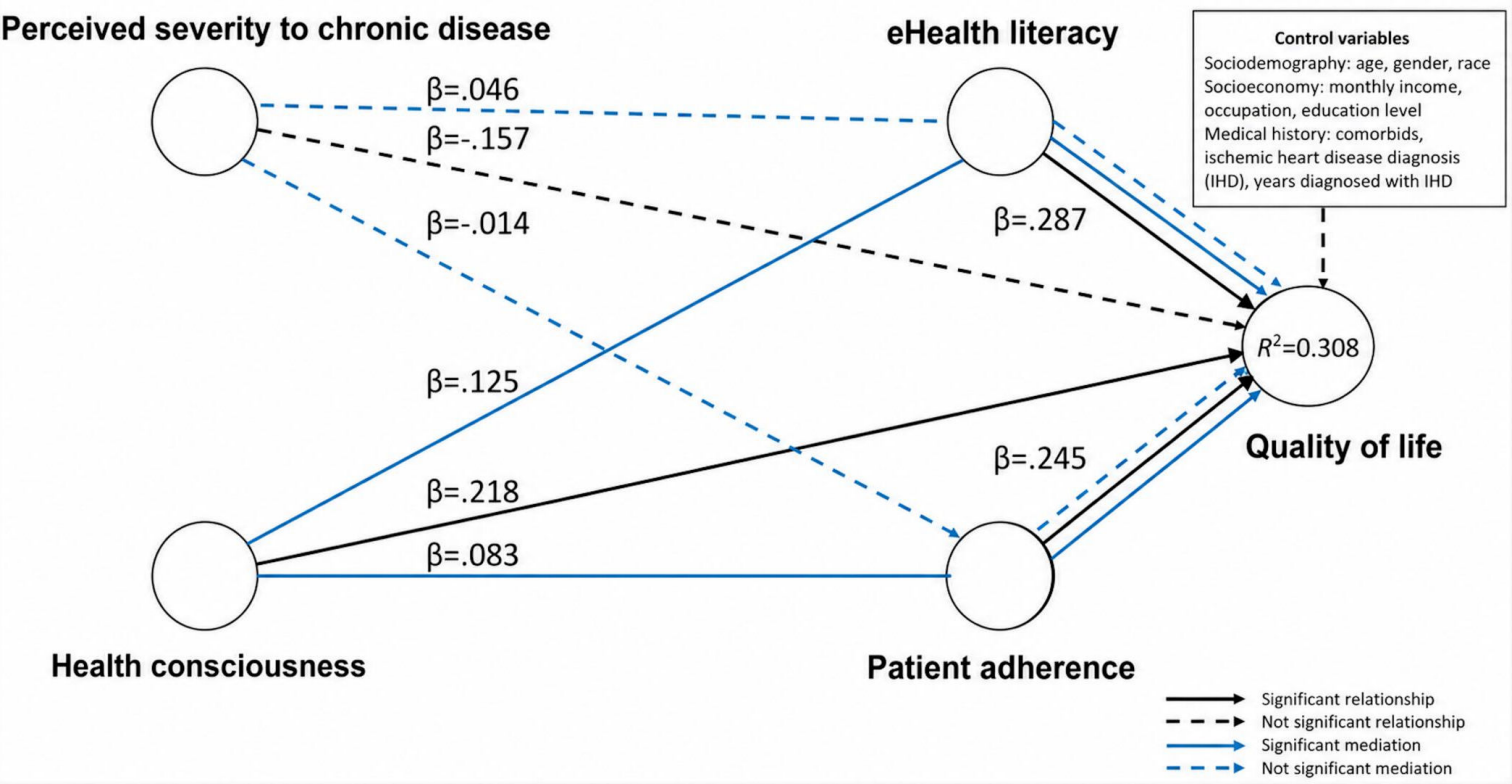
Final parallel mediation pathway model derived from the partial least squares-structural equation modeling (PLS-SEM) analysis among young patients with ischemic heart disease attending tertiary cardiac referral centers in Klang Valley, Malaysia (November 2021-June 2022), showing statistically significant pathways predicting quality of life.

The parallel mediation model demonstrated medium predictive power, considering that the MAE values from PLS structural equation modeling were lower compared to those of the LM for most construct items. In contrast, the serial mediation model exhibited low predictive power, given that the MAE values of PLS structural equation modeling were only lower than those of the LM for a limited number of construct items. Figures3  4 present the final models, illustrating the relationships via parallel and serial mediation models.

**Figure 4.**
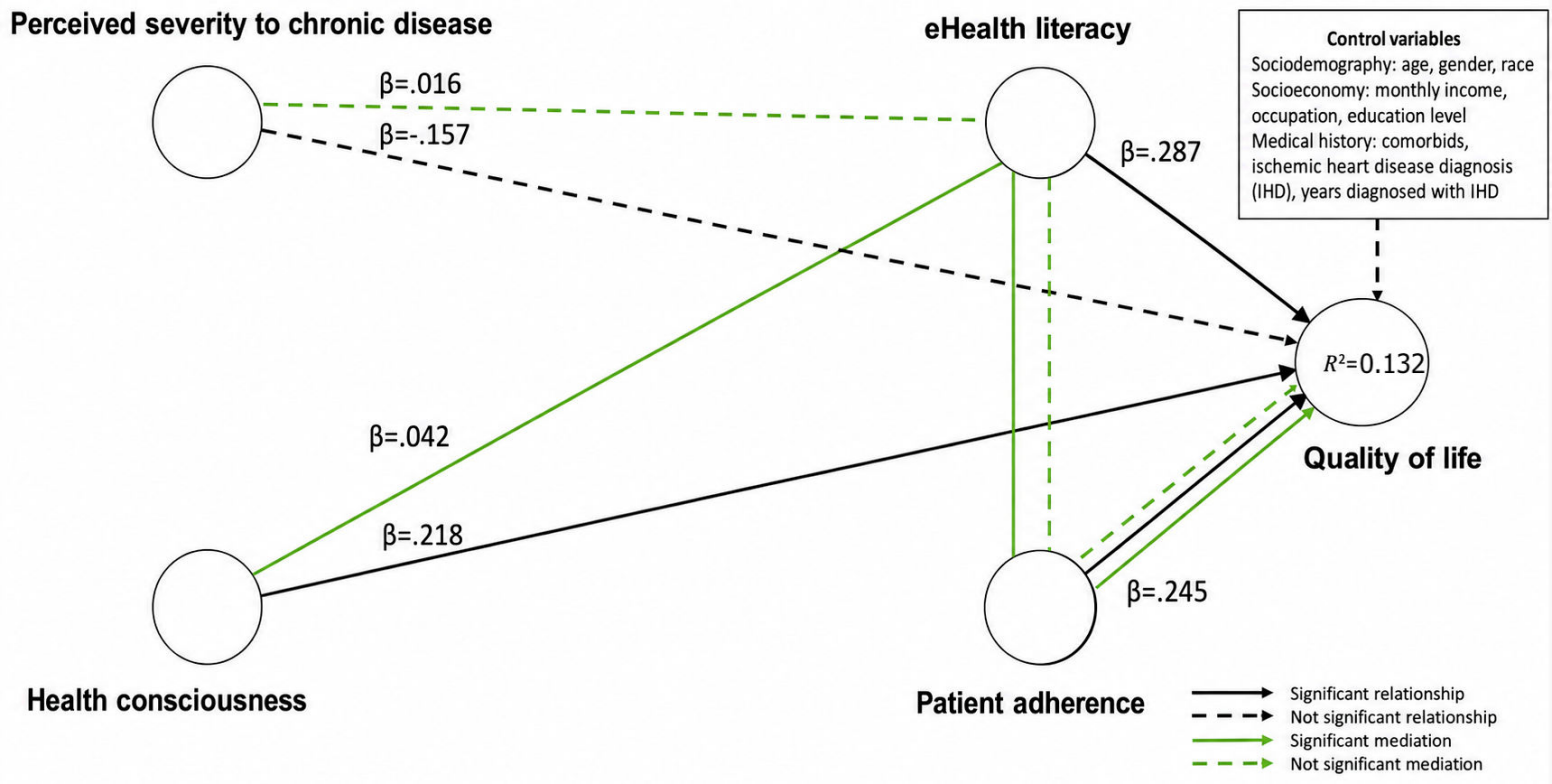
Final serial mediation pathway model using the partial least squares-structural equation modeling (PLS-SEM) analysis from a cross-sectional study among young patients with ischemic heart disease in Klang Valley, Malaysia (November 2021-June 2022), illustrating sequential mediation effects of eHealth literacy and patient adherence on quality-of-life outcomes.

## Discussion

### Findings

The model examined in this study was grounded in the TMeHL [17], which focuses on the effects of eHealth literacy, PA, and other factors on QoL. An investigation of the model, which explored both direct and mediation effects, revealed that eHealth literacy was the most significant factor influencing QoL in young patients with IHD, exceeding the impact of HC and PA, whereas the PS had no considerable effect. This study highlights an important and previously underexplored aspect of eHealth literacy, examining its interplay with other factors influencing QoL. The TMeHL theory offers a robust framework for understanding the dynamic and complex interactions between individuals and eHealth technologies, providing valuable insights into this evolving domain.

The role of HC in the direct pathway hypothesis, “H1: Health consciousness is positively and significantly related to quality of life,” was supported in this study. HC was significantly related to the QoL of young patients with IHD, influencing both direct and indirect pathways in the parallel and serial mediation models.

The role of eHealth literacy was also supported by multiple hypotheses. The direct pathway hypothesis, “H4: eHealth literacy is positively and significantly related to quality of life,” the parallel mediation hypothesis, “H6: eHealth literacy positively mediates the relationship between health consciousness and quality of life in a parallel pathway,” and the serial mediation hypothesis, “H9: eHealth literacy and patient adherence positively serially mediate the relationship between health consciousness and quality of life,” were validated by the findings. The results highlight the key role eHealth literacy plays in enhancing the QoL of young individuals with IHD.

The findings highlight the significant role of eHealth literacy in enhancing QoL, aligning with existing but limited research indicating its potential to empower individuals to make informed health decisions [54]. Notably, incorporating eHealth literacy as a mediator between HC and QoL adds considerable value, as demonstrated by the parallel mediation model. Individuals with higher eHEALS scores are often “information explorers” capable of identifying reliable information sources and resolving conflicting information with minimal assistance [55].

The observed levels of eHealth literacy scores may reflect broader contextual factors during the study period, including reliance on digital health information; however, this study did not directly measure such influences [56]. Proficiency in eHealth literacy enables young patients with IHD to manage overwhelming health information effectively, facilitating better decision-making, social support, and access to health care services, thereby improving QoL [57]. The pandemic’s “infodemic” further underscores the critical need for reliable health information and the empowerment of patients with eHealth skills [56].

The final models demonstrated that eHealth literacy significantly contributes, both directly and indirectly, to improving QoL. This underscores its importance as a foundation for enhancing self-care among young patients with IHD through the effective use of technology, ultimately fostering better health outcomes and a greater QoL.

The study revealed a negative direct effect between PS, also referred to as illness perceptions, and QoL. Perceived severity influences distress levels, which ultimately affect QoL outcomes [58]. This construct is associated with various factors, including physical functioning, disability, depression, and anxiety. Notably, contemporary researchers applying the Health Belief Model often overlook emotional aspects when defining severity [59]. According to the Health Belief Model, perceived vulnerability and disease severity together form a “threat” that drives action [60]. Actions are further influenced by an individual’s beliefs about the availability and efficacy of treatments or solutions. When both perceived vulnerability and severity are absent, there is no motivation to act.

Participants in this study demonstrated perceptions consistent with viewing IHD as a chronic condition; however, temporal changes in illness perception cannot be inferred due to the cross-sectional design. A deeper understanding of IHD often contributes to a prolonged perception of the illness and increased concern. The diagnosis of a chronic disease imposes significant physical, emotional, and social burdens on young patients, affecting their overall environment over time. In conclusion, as young patients with IHD transition from perceiving their illness as acute to viewing it as a chronic condition, they develop a more negative perception. This shift stems from the demands of lifelong treatment, regular follow-up, and preventive measures, which can exacerbate emotional and psychological challenges over time.

Effective eHealth literacy is vital for accessing reliable health information, which can enhance patients’ adherence to medical recommendations among young patients with IHD. This adherence encompasses critical aspects, such as medication, lifestyle modifications, and dietary changes. The importance of PA in the model is underscored by its dual role, having both direct and mediating effects on QoL. Following medical advice and recommendations significantly contributes to a better QoL for young patients with IHD.

Moreover, eHealth literacy plays a pivotal role in improving PA by enabling patients to access trustworthy health information and apply it effectively. By fostering patients’ adherence to health advice tailored to their condition, eHealth literacy supports young patients with IHD in managing their disease and enhancing their QoL.

The final model of this study aligns with the theoretical foundation of the TMeHL, as the parallel mediation structure reflects the dynamic and transactional nature of eHealth [17]. The parallel model incorporates three key assumptions: (1) task-oriented and user-oriented factors, such as HC and individual perceptions; (2) eHealth literacy, which represents a multifaceted and hierarchical set of intrapersonal skills; and (3) patient engagement, demonstrated through PA, which results from being informed and empowered, ultimately affecting health outcomes.

In practice, parallel mediation, where multiple tasks are addressed simultaneously, is more feasible and practical than serial mediation, where tasks are completed sequentially. Improving the QoL of young patients with IHD in the digital age necessitates promoting HC and eHealth literacy while fostering PA to medical recommendations. Limited health literacy has been associated with inadequate understanding of illnesses, poor disease management, increased hospitalization rates, and reduced use of preventive services.

The COVID-19 pandemic has emphasized the crucial role of eHealth literacy in health care delivery, with eHealth resources serving as vital tools for linking care and enhancing resilience. Moreover, the concept of capability is key to striking a balance between autonomy and paternalism, empowering individuals to make informed health care decisions while safeguarding them from harm [54]. This approach highlights the need to equip young patients with IHD with the tools and knowledge necessary for optimal self-care and improved health outcomes.

### Strengths and Limitations

This study’s novelty lies in its integration of the TMeHL theory [17] to explore eHealth literacy among young patients with IHD. This is the first study to utilize this model, highlighting the growing importance of eHealth literacy, as young patients increasingly engage with online health care resources. This study aims to provide a thorough analysis of the elements that influence eHealth literacy and how it impacts QoL. Another strength is the focus on young patients with IHD, a demographic experiencing a growing prevalence of IHD both locally and globally. This approach provides insights into unique factors influencing IHD in younger populations, including the potential role of eHealth literacy in early detection and disease management.

One limitation of the study is its cross-sectional design; therefore, causal relationships and temporal directionality among constructs cannot be established. Additionally, the use of consecutive sampling, although practical, is nonrandom and may limit generalizability. Given that the sample size depended on patient accessibility and the order of enrollment, the sample might not provide a comprehensive representation of the wider young patient population with IHD. This study faced challenges, such as restricted in-person data collection, limited site access, and movement restrictions under the Movement Control Order during the COVID-19 pandemic. Additional obstacles included participants’ COVID-19 status (eg, positive cases and persons under investigation) and time constraints requiring a focus on stable patients. Online data collection methods, including virtual interviews, mitigated some challenges but introduced issues, such as unequal technology access, potential selection bias, and lower response rates. The pandemic’s dynamic nature has also had confounding effects on QoL assessment, necessitating cautious generalization of findings.

### Conclusion and Recommendations

The study emphasized the importance of a parallel mediating relationship between eHealth literacy and PA, driven by HC, in predicting QoL improvements. These findings align with the TMeHL theory, confirming reliable and valid constructs with medium predictive power for understanding and enhancing QoL among young patients with IHD. Future research may explore whether digital tools, structured eHealth education, or provider-led interventions can strengthen these relationships.

eHealth literacy is crucial for young patients with IHD in the digital health era, as it enables them to access trustworthy online resources, critically evaluate health information, and communicate effectively with health care providers via telemedicine. The incorporation of eHealth literacy into daily life facilitates self-management tasks, such as symptom tracking and medication adherence. In Malaysia, apps such as MyHealth and MySejahtera serve as credible platforms to promote health education and improve digital health skills. Certification programs could further validate and enhance eHealth literacy competencies.

Integrating eHealth literacy with PA can greatly enhance QoL, particularly in areas such as physical health, where challenges, such as pain, often disrupt daily activities. Health care providers play a pivotal role by introducing digital tools and educating patients on their effective use for medication compliance, pain management, and lifestyle modifications.

Interactive digital resources further empower patients by providing secure platforms for sharing information and communicating with health care teams. By adopting these approaches, young patients with IHD can achieve better disease management, improved health outcomes, and enhanced QoL.

## Supplementary material

10.2196/71647 Multimedia Appendix 1 Hypothesis testing direct effects.

10.2196/71647 Multimedia Appendix 2 Hypothesis testing indirect effects.

10.2196/71647 Multimedia Appendix 3 Assessment of full collinearity testing.

10.2196/71647 Multimedia Appendix 4 Outer loadings, convergent validity, and internal consistency reliability of the constructs.

10.2196/71647 Checklist 1 STROBE checklist.

## Abbreviations

BCI LL: bias-corrected CI for lower limit
BCI UL: bias-corrected CI for upper limit
CVD: cardiovascular disease
eHEALS: eHealth Literacy Scale
eHealth: eHealth literacy
HC: health consciousness
IHD: ischemic heart disease
LM: linear model
MAE: mean absolute error
PA: patient adherence
PLS: partial least squares
PS: perceived severity to chronic disease
QOL: quality of life
STEMI: ST-elevation myocardial infarction
STROBE: Strengthening the Reporting of Observational Studies in Epidemiology
TMeHL: Transactional Model of eHealth Literacy
WHO: World Health Organization
