# Supplementary material for: The Role of eHealth Literacy and Patient Adherence in Mediating Health Consciousness and Perceived Severity in Quality of Life Among Young Patients With Ischemic Heart Disease: Cross-Sectional Study
Source: JMIR Form Res. 2026 May 26;10:e71647. doi: 10.2196/71647 (PMC13211866; doi:10.2196/71647)
Supplement: Multimedia Appendix 1 [file formative-v10-e71647-s001.docx]

Table S3. Hypothesis testing of direct effects within the structural model derived from a cross-sectional study among young patients with ischemic heart disease in tertiary cardiac referral centers in Klang Valley, Malaysia (November 2021–June 2022; N=136), examining relationships between eHealth literacy, Patient adherence, Health consciousness, Perceived severity to chronic disease, and Quality of life using partial least squares structural equation modeling (PLS-SEM).

| Hypothesis | Relationship | Std. Beta, β | Std. Dev. | *t*-  values | *P* values | BCI LL | BCI UL | *f^2^* | R^2^ | Quantification of effect size (*f^2^*) | Interpretation |  |
| --- | --- | --- | --- | --- | --- | --- | --- | --- | --- | --- | --- | --- |
| H1 | Health consciousness -> Quality of life | 0.218 | 0.103 | 2.114 | .017 | 0.050 | 0.388 | 0.038 | 0.308 | Small | Hypothesis supported |  |
| H2 | Perceived severity to chronic disease -> Quality of life | -0.157 | 0.102 | 1.543 | .061 | -0.325 | 0.007 | 0.025 |  | Small | Hypothesis not supported |  |
| H3 | Patient adherence -> Quality of life | 0.245 | 0.116 | 2.123 | .017 | 0.034 | 0.418 | 0.066 |  | Small | Hypothesis supported |  |
| H4 | eHealth literacy -> Quality of life | 0.287 | 0.099 | 2.887 | .002 | 0.127 | 0.453 | 0.075 |  | Small | Hypothesis supported |  |
| Control variables | | | | | | | | | | | | |
|  | Age -> Quality of life | 0.088 | 0.089 | 0.989 | .161 | -0.069 | 0.226 | 0.009 |  | None |  |  |
|  | Comorbids -> Quality of life | -0.261 | 0.181 | 1.439 | .075 | -0.551 | 0.049 | 0.017 |  | None |  |  |
|  | Education -> Quality of life | -0.194 | 0.184 | 1.052 | .146 | -0.487 | 0.115 | 0.009 |  | None |  |  |
|  | Gender -> Quality of life | -0.163 | 0.214 | 0.764 | .223 | -0.501 | 0.198 | 0.004 |  | None |  |  |
|  | Ischemic heart disease diagnosis -> Quality of life | 0.058 | 0.191 | 0.302 | .381 | -0.282 | 0.343 | 0.001 |  | None |  |  |
|  | Monthly income -> Quality of life | 0.165 | 0.110 | 1.509 | .066 | -0.007 | 0.355 | 0.022 |  | Small |  |  |
|  | Occupation -> Quality of life | -0.296 | 0.238 | 1.245 | .107 | -0.678 | 0.106 | 0.011 |  | None |  |  |
|  | Race -> Quality of life | 0.127 | 0.202 | 0.630 | .264 | -0.200 | 0.458 | 0.004 |  | None |  |  |
|  | Years diagnosed with Ischemic heart disease -> Quality of life | 0.127 | 0.170 | 0.745 | .228 | -0.173 | 0.391 | 0.005 |  | None |  |  |

Note: 95% confidence interval was used with a bootstrapping of 5000

Footnote: BCI LL: bias-corrected confidence intervals for lower limits; BCI UL: bias-corrected confidence intervals for upper limits
