# Supplementary material for: The Role of eHealth Literacy and Patient Adherence in Mediating Health Consciousness and Perceived Severity in Quality of Life Among Young Patients With Ischemic Heart Disease: Cross-Sectional Study
Source: JMIR Form Res. 2026 May 26;10:e71647. doi: 10.2196/71647 (PMC13211866; doi:10.2196/71647)
Supplement: Multimedia Appendix 2 [file formative-v10-e71647-s002.docx]

Table S4. Hypothesis Testing of indirect effects within the structural model derived from a cross-sectional study among young patients with ischemic heart disease in tertiary cardiac referral centers in Klang Valley, Malaysia (November 2021–June 2022; N=136), examining relationships between eHealth literacy, Patient adherence, Health consciousness, Perceived severity to chronic disease, and Quality of life using partial least squares structural equation modeling (PLS-SEM).

| Hypothesis | Relationship | Std. Beta | Std. Dev. | *t*- values | *P* values | BCI LL | BCI UL | *f^2^* | Quantification of effect size (*f^2^*) | Interpretation |
| --- | --- | --- | --- | --- | --- | --- | --- | --- | --- | --- |
|  | Parallel mediation model | | | | | | | | | |
| H5 | Health consciousness -> Patient adherence -> Quality of life | 0.083 | 0.049 | 1.687 | .046 | 0.016 | 0.178 | 0.007 | None | Hypothesis supported |
| H6 | Health consciousness -> eHealth literacy -> Quality of life | 0.125 | 0.052 | 2.403 | .008 | 0.059 | 0.241 | 0.016 | Small | Hypothesis supported |
| H7 | Perceived severity to chronic disease -> Patient adherence -> Quality of life | -0.014 | 0.030 | 0.461 | .322 | -0.077 | 0.021 | 0.000 | None | Hypothesis not supported |
| H8 | Perceived severity to chronic disease -> eHealth literacy -> Quality of life | 0.046 | 0.038 | 1.219 | .111 | 0.001 | 0.121 | 0.002 | None | Hypothesis not supported |
|  | Serial mediation model | | | | | | | | | |
| H9 | Health consciousness -> eHealth literacy -> Patient adherence -> Quality of life | 0.042 | 0.025 | 1.653 | .049 | 0.012 | 0.097 | 0.002 | None | Hypothesis supported |
| H10 | Perceived severity to chronic disease -> eHealth literacy -> Patient adherence -> Quality of life | 0.016 | 0.015 | 1.037 | .150 | 0.000 | 0.046 | 0.000 | None | Hypothesis not supported |

Note: 95% confidence interval was used with a bootstrapping of 5000

Footnote: BCI LL: bias-corrected confidence intervals for lower limits; BCI UL: bias-corrected confidence intervals for upper limits
