# Supplementary material for: The Role of eHealth Literacy and Patient Adherence in Mediating Health Consciousness and Perceived Severity in Quality of Life Among Young Patients With Ischemic Heart Disease: Cross-Sectional Study
Source: JMIR Form Res. 2026 May 26;10:e71647. doi: 10.2196/71647 (PMC13211866; doi:10.2196/71647)
Supplement: Multimedia Appendix 4 [file formative-v10-e71647-s004.docx]

Table S2. Outer loadings (indicator reliability), convergent validity (average variance extracted, AVE) and internal consistency reliability (composite reliability, CR) of the constructs from a cross-sectional study examining the relationship between eHealth literacy, Patient adherence, Health consciousness, Perceived severity to chronic disease, and Quality of life among young patients with ischemic heart disease attending tertiary cardiac referral centers in the Klang Valley, Malaysia (November 2021–June 2022; N=136), analyzed using partial least squares structural equation modeling (PLS-SEM).

| Measurement | Item | Loadings | AVE | CR |
| --- | --- | --- | --- | --- |
|  |  |  |  |  |
| Health consciousness | H1 | 0.900 | 0.823 | 0.959 |
|  | H2 | 0.937 |  |  |
|  | H3 | 0.895 |  |  |
|  | H4 | 0.911 |  |  |
|  | H5 | 0.891 |  |  |
| Perceived severity to chronic disease | P1 | 0.697 | 0.632 | 0.873 |
|  | P2 | 0.820 |  |  |
|  | P3 | 0.822 |  |  |
|  | P4 | 0.834 |  |  |
| eHealth literacy | E1 | 0.901 | 0.778 | 0.965 |
|  | E2 | 0.915 |  |  |
|  | E3 | 0.904 |  |  |
|  | E4 | 0.901 |  |  |
|  | E5 | 0.861 |  |  |
|  | E6 | 0.914 |  |  |
|  | E7 | 0.818 |  |  |
|  | E8 | 0.836 |  |  |
| Patient adherence | Behaviors | 0.936 | 0.661 | 0.792 |
|  | Recommendations | 0.668 |  |  |
| Quality of life | Environment | 0.916 | 0.751 | 0.923 |
|  | Physical health | 0.775 |  |  |
|  | Psychological | 0.843 |  |  |
|  | Social relationship | 0.925 |  |  |

Footnote: AVE: average variance extracted; CR: composite reliability; P: Perceived severity to chronic disease; H: Health consciousness; E: eHealth literacy
